# An Unusual Presentation of Tuberculosis With Dysphagia

**DOI:** 10.7759/cureus.30174

**Published:** 2022-10-11

**Authors:** Ananta Subedi, Rakshya Sharma

**Affiliations:** 1 Internal Medicine, Avera McKennan Hospital & University Health Center, Sioux Falls, USA; 2 Internal Medicine, University of South Dakota Sanford School of Medicine, Sioux Falls, USA

**Keywords:** tb, tuberculosis, pott disease, unusual presentation, dysphagia, tuberculosis spondylitis

## Abstract

About a quarter of the world’s population is infected with tuberculosis (TB). It is one of the leading causes of death worldwide. However, the prevalence of TB in the United States is rare. Pulmonary TB is the commonest form of TB. Most patients with TB present with pulmonary symptoms. Extrapulmonary TB usually presents with symptoms related to the organ system involved and can present with very unusual symptoms. TB presenting with dysphagia is uncommon, and spinal TB presenting with dysphagia is very unusual.

A 57-year-old woman presented to the emergency department with a four-month history of dysphagia and chest pain. She was undergoing an outpatient workup of her symptoms that included esophagogastroduodenoscopy, gastric emptying study, and computed tomography (CT) scan of the chest, which showed incidental findings of a focus in the thoracic spine. It was followed by a bone scan, and the results were concerning for malignancy. She was awaiting an oncology appointment when she presented to us. A basic workup after her presentation that includes complete blood count, comprehensive metabolic panel, troponin, chest x-ray, and electrocardiogram was unrevealing. Magnetic resonance imaging (MRI) of the thoracic spine showed findings suggestive of tuberculous spondylitis, tuberculous paraspinal, and prevertebral abscesses. Chest CT was repeated, which showed mass effect and erosion on the posterior esophageal wall with anterior displacement of the esophagus. Tissue biopsy revealed acid-fast bacilli (AFB) on AFB stain, and the culture grew *Mycobacterium tuberculosis*. She was successfully treated with the antitubercular regimen of rifampin, isoniazid, ethambutol, and pyrazinamide.

TB can present with a myriad of symptoms, and although rare, it can present with symptoms like dysphagia. In patients with a history of travel to or immigration from an endemic region, previous infection, and immunosuppression, TB should be considered as one of the differential diagnoses even for unusual symptoms like dysphagia.

## Introduction

Tuberculosis (TB) is one of the leading causes of death worldwide. About a quarter of the global population is infected with *Mycobacterium tuberculosis *[1]. In 2020, 4.8 million people were diagnosed with pulmonary TB globally [2]. However, the incidence of TB in the United States is low. Hence, the diagnosis of TB can be frequently missed or delayed, which is especially true with extrapulmonary TB. Here, we present a case of extrapulmonary TB, which had an unusual presentation. An extensive workup was necessary to reach the correct diagnosis of extrapulmonary TB and to eventually initiate the appropriate treatment.

## Case presentation

A 57-year-old woman with a medical history of diabetes mellitus and hypertension presented to the emergency department with complaints of dysphagia and burning chest pain. She had similar complaints for about four months before her presentation, but her symptoms were progressively worsening. She also had back pain for about the same duration. She had extensive workup done as an outpatient for her symptoms, which included a complete blood count (CBC), comprehensive metabolic panel (CMP), electrocardiogram, chest x-ray, abdominal x-ray, and x-ray of the thoracic spine; all were within normal limits. She was referred to a gastroenterology clinic for dysphagia, and she had an esophagogastroduodenoscopy and gastric emptying study done, which were unremarkable. She was placed on a proton pump inhibitor (PPI) and sucralfate but had no relief.

Two weeks before her presentation, she had presented to urgent care with chest pain and back pain. Electrocardiogram and troponins were negative. She had a computed tomography (CT) of the chest done, which showed a lucent focus in the anterior vertebral body in T6. This finding was followed up with a bone scan that confirmed the lesion. The new lesion was concerning for malignancy, and she had an appointment with oncology upcoming. CT of the chest, abdomen, and pelvis did not show any suspicious lytic lesions in the bones or any findings concerning of acute pathology in the abdomen and pelvis. On further inquiry, she disclosed further symptoms that include anorexia, unintentional weight loss, night sweats, and dry cough. Her pertinent social history included immigration to the United States from South Asia in 2012.

**Figure 1:**
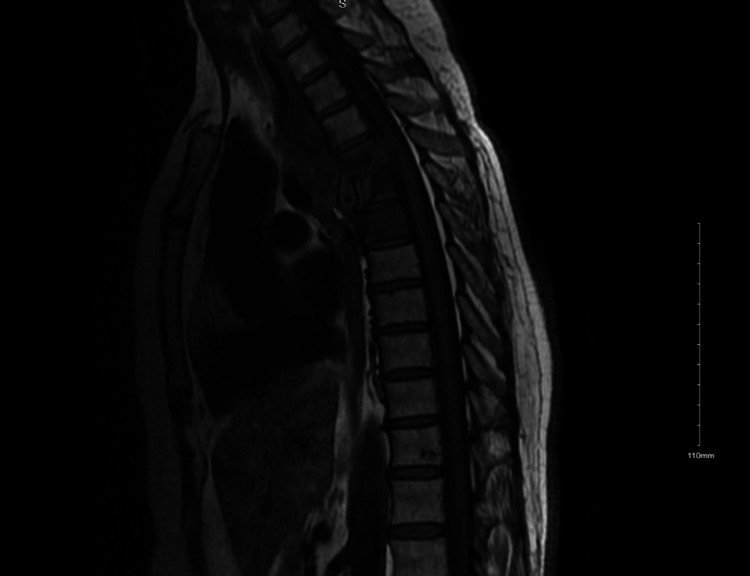
MRI of the thoracic spine

Her physical exam on presentation was fairly unremarkable except for minimal paraspinal tenderness. Initial CBC and CMP were within normal limits. Troponins were negative twice. An electrocardiogram and chest x-ray did not show any acute pathology. Magnetic resonance imaging (MRI) of the thoracic spine was done, which showed abnormal marrow signals involving T4, T5, and T6 with disc narrowing as well as end plate destruction at T4-T5 and multiloculated paraspinal and posterior mediastinal mass or collection, suggestive of tuberculous spondylitis with tuberculous paraspinal and prevertebral abscess (Figure 1).

**Figure 2:**
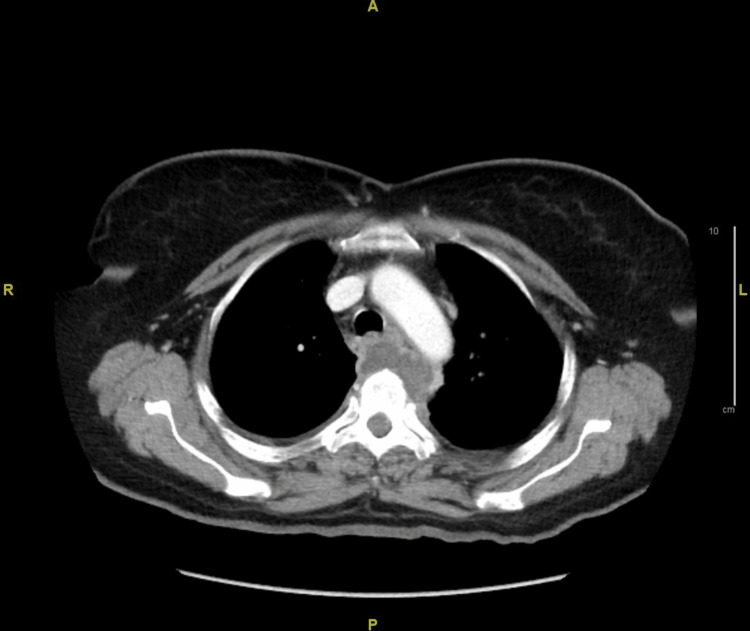
CT scan of the chest

After the MRI findings, on a further query, the patient revealed that she had a history of untreated latent TB. An acid-fast bacillus (AFB) stain of early morning sputum samples was done, which was negative. CT-guided aspiration of the paraspinal abscess was drawn and sent for a workup. While in the hospital, her dysphagia further worsened. Repeat chest CT with contrast was done. It showed mass effect and erosion on the posterior esophageal wall anterior to the T3 and T4 vertebra with anterior displacement of the esophagus (Figure 2). This finding explained her dysphagia.

Bone biopsy and the drained abscess were positive for AFB stain and later also grew *M. tuberculosis*. With her diagnosis of extrapulmonary TB, she was started on antitubercular treatment with rifampin, isoniazid, ethambutol, and pyrazinamide. Neurosurgery recommended cervical thoracic orthosis brace and physical therapy. She was then discharged from the hospital. Her symptoms had resolved in her nine-month follow-up after discharge.

## Discussion

TB is a communicable disease caused by *M. tuberculosis*. Before the SARS-CoV-2 pandemic, TB was the leading cause of infectious disease-related mortality worldwide [2]. About a quarter of the global population is expected to be infected with *M. tuberculosis* [1]. TB primarily affects the lungs, but it can affect any body system. An estimated 15%-20% of TB cases are extrapulmonary. About 10% of extrapulmonary TB is skeletal, and about 50% of skeletal TB involves the spine [3].

Tuberculous spondylitis, also known as Pott disease, is a TB infection of the spine. Bacillemia and hematogenous spread of *M. tuberculosis* to the cancellous bone of the vertebral bodies results in the spinal involvement of TB. The most common primary site of infection is either pulmonary or infection of the genitourinary system [4]. Spinal TB usually presents as local pain, tenderness, muscle spasms, cold abscess, and spinal deformity. Constitutional symptoms like fever, weight loss, anorexia, and night sweats might also be present. The progression of spinal TB is slow and insidious, and patients usually present late in the disease process only when there is severe pain, spinal deformity, or neurological symptoms. Neurological symptoms are common and may progress to paraplegia or quadriplegia, if left untreated [5].

It is very uncommon for TB to present with dysphagia. Primary esophageal TB is very rare because of different protective factors of the esophagus that include stratified squamous epithelium, esophageal peristalsis, saliva, and erect posture [6]. Hence, most cases of dysphagia in TB are because of the secondary involvement of the esophagus due to the surrounding infected structures like peri-esophageal lymph nodes, mediastinal lymph nodes, pulmonary sites, larynx, pharynx, or spine [7]. Dysphagia as an uncommon symptom of spinal TB has been mentioned in the literature mostly as a consequence of cervical spine TB because of cold abscess or retropharyngeal abscess [5-7]. We could not find any reported cases where dysphagia was the primary complaint in a patient with spinal TB or any cases where dysphagia was a complaint in a patient with thoracic spinal TB.

Diagnosis of skeletal TB is challenging because of the indolent course of the disease and the absence of pulmonary involvement. Comprehensive history that includes questions about the country of origin, previous history of TB, or history of close contact with a patient with TB as well as a history of immunosuppression can provide an important clue. Characteristics of radiological findings in CT scans and MRI also aid in the diagnosis. Confirmatory diagnosis is established by the demonstration of acid-fast bacilli on microscopy and culture of material obtained following either the biopsy of the lesion or needle aspiration of the infected tissue [8]. This is followed by drug susceptibility testing.

The mainstay of treatment of spinal TB is chemotherapy with multiple antitubercular medications. The first-line oral agents are rifampin, isoniazid, pyrazinamide, ethambutol, and rifabutin. World Health Organization (WHO) and Centers for Disease Control and Prevention (CDC) recommend two months of rifampin, isoniazid, ethambutol, and pyrazinamide followed by a four-month of rifampin and isoniazid. Compliance with the medication regimen is of paramount importance to prevent relapse and resistance. Surgical management of spinal TB is recommended if there is a significant deformity or a neurological deficit [5].

TB is rare in non-endemic regions like the United States, and extrapulmonary TB is even rarer. Prompt diagnosis and appropriate treatment can prevent complications that can include paraplegia and death. Timely diagnosis is also important in the epidemiological aspect as it can help prevent transmission of the disease [4].

In our case, the primary complaint of the patient was dysphagia and burning chest pain. Outpatient workup prior to admission was geared toward gastroesophageal reflux disease (GERD) and common causes of dysphagia as well as ruling out the common causes of chest pain, mainly cardiopulmonary etiologies. After CT of the chest showed a lucent focus on the T6 vertebra, malignancy was the working diagnosis. A considerable amount of time and resources were spent before reaching the correct diagnosis. Only after further imaging was done and after a more comprehensive history was obtained, the correct diagnosis was suspected. Fortunately, she received treatment before attaining adverse outcomes like paraplegia and death and eventually recovered back to her usual health.

## Conclusions

TB is rare in the United States, and spinal TB is even rarer. TB can affect any organ system, but it is very unusual for TB to present with dysphagia. Hence, the diagnosis can be easily missed. TB should be among the differentials in appropriate cases with pertinent history such as immigration from an endemic country, history of travel to endemic countries, history of prior infection, history of immunosuppression, etc. Timely diagnosis and treatment of such cases can prevent substantial morbidity and mortality.
